# Inhibition of carbohydrate digestive enzymes by a complementary essential oil blend: in silico and mixture design approaches

**DOI:** 10.3389/fphar.2025.1522124

**Published:** 2025-03-10

**Authors:** El Hassania Loukili, Mouhcine Fadil, Amal Elrherabi, Mohammed Er-rajy, Mohamed Taibi, Khalil Azzaoui, Rachid Salghi, Rachid Sabbahi, Mohammed M. Alanazi, Larbi Rhazi, Aleksandar Széchenyi, Mohamed Siaj, Belkheir Hammouti

**Affiliations:** ^1^ Euromed University of Fes, UMEF, Fes, Morocco; ^2^ Laboratory of Applied Organic Chemistry, Faculty of Sciences and Techniques, Sidi Mohamed Ben Abdellah University, Fez, Morocco; ^3^ Laboratory of Bioresources, Biotechnology, Ethnopharmacology and Health, Faculty of Sciences, University Mohammed 1st, Oujda, Morocco; ^4^ LIMAS Laboratory, Faculty of Sciences Dhar El Mahraz, Sidi Mohamed Ben Abdellah University, Fez, Morocco; ^5^ Laboratoire d’Amélioration des Productions Agricoles, Biotechnologie et Environnement (LAPABE), Faculty of Sciences, Mohammed First University, Oujda, Morocco; ^6^ Engineering Laboratory of Organometallic, Molecular Materials and Environment, Faculty of Sciences, Sidi Mohamed Ben Abdellah University, Fez, Morocco; ^7^ Laboratory of Industrial Engineering, Energy and the Environment (LI3E) SUPMTI, Rabat, Morocco; ^8^ Laboratory of Applied Chemistry and Environment, ENSA, University Ibn Zohr, Agadir, Morocco; ^9^ Research Team in Science and Technology, Higher School of Technology of Laayoune, Ibn Zohr University, Laayoune, Morocco; ^10^ Department of Pharmaceutical Chemistry, College of Pharmacy, King Saud University, Riyadh, Saudi Arabia; ^11^ Institut Polytechnique UniLaSalle, Université d’Artois, Beauvais, France; ^12^ Institute of Pharmaceutical Technology and Biopharmacy, Faculty of Pharmacy, University of Pécs, Pécs, Hungary; ^13^ Green Chemistry Research Group, János Szentágothai Research Centre, University of Pécs, Pécs, Hungary; ^14^ Department of Chemical Engineering and Biotechnological Engineering, Université de Sherbrooke, Sherbrooke, QC, Canada

**Keywords:** DFT, diabetes management, molecular docking, volatile substances, pancreatic-α-amylase, intestinal-α-glucosidase

## Abstract

**Background:**

The increasing demand for natural alternatives in diabetes treatment has driven research into plant-derived metabolites, particularly essential oils (EOs) with bioactive properties. This study aims to optimize an EO mixture for inhibiting two key enzymes involved in glucose digestion: pancreatic α-amylase and intestinal α-glucosidase.

**Methods:**

Essential oils were extracted from three Moroccan medicinal plants: false yellowhead (*Inula viscosa* L.), rose geranium (*Pelargonium graveolens* L'Hér.), and lemongrass (*Cymbopogon citratus* (DC.) Stapf.). Gas chromatography-mass spectrometry (GC-MS) analysis identified key metabolites in each EO. A statistical mixture design was employed to evaluate different EO ratios for their inhibitory effects on α-amylase and α-glucosidase. Additionally, density functional theory (DFT) calculations and molecular docking simulations were conducted to assess the key metabolites' electronic properties and interaction potential with target enzymes.

**Results:**

GC-MS analysis identified 32 metabolites in *P*. *graveolens*, with citronellol (18.67%), eucalyptol (13.30%), and 2-octen-1-ol (8.12%) as major components. *I*. *viscosa* contained 18 metabolites, dominated by 2-camphanol acetate (51.12%) and camphol (19.32%), while *C. citratus* had 23 metabolites, with α-citral (24.70%) and 2-isopropenyl-5-methylhex-4-enal (29.25%) as key constituents. The optimal formulation for α-glucosidase inhibition was a binary mixture of 73% *C. citratus* and 27% *P. graveolens*. In contrast, the best blend for α-amylase inhibition consisted of 56% *P. graveolens* and 44% *I. viscosa*. DFT calculations confirmed the electrophilic nature of key metabolites, supporting their potential for enzyme interaction. Molecular docking simulations suggested that these phytochemicals could exhibit stronger inhibitory effects than acarbose, a widely used antidiabetic drug.

**Conclusion:**

These findings highlight the potential of optimized EO formulations as natural alternatives for managing hyperglycemia and developing novel diabetes therapies.

## 1 Introduction

For millennia, civilizations have relied on medicinal plants for their therapeutic, cosmetic, nutritional, pharmaceutical, and industrial properties (Lahsissene et al., 2009; Azzaoui et al., 2022). In recent years, there has been growing interest in medicinal and aromatic plants (Loukili et al., 2022b; Loukili et al., 2024a) due to their effectiveness, ease of extraction, and the hazards associated with synthetic chemicals (Taibi et al., 2024a; Nouioura et al., 2024). Many studies have focused on developing high-quality extracts from these plants, and their biological activity is a key indicator of their effectiveness (El Youssfi et al., 2024; Haddou M. et al., 2023).

Among these plant-derived products, essential oils (EOs), concentrated extracts from aromatic plants, are particularly valued for diverse bioactive properties, including antidiabetic activity (El Guerrouj et al., 2023; Taibi et al., 2024e). They are becoming increasingly popular in commercial and scientific research due to their antioxidant, antibacterial, antifungal, and antidiabetic properties (Dung et al., 2008; Taibi et al., 2024c). The antidiabetic potential of EOs, particularly for treating metabolic disorders such as type 2 diabetes, has attracted significant attention (Ouahabi et al., 2024). EOs are rich in bioactive metabolites, such as terpenes, phenols, and monoterpenes, which have been shown to regulate blood glucose, enhance insulin sensitivity, and reduce oxidative stress (Elbouzidi et al., 2024a; Hbika et al., 2024).

The global rise in metabolic diseases like diabetes underscores the need for novel, safer, and more natural treatment approaches. While synthetic enzyme inhibitors are commonly used to manage these disorders, they often cause adverse side effects, highlighting the importance of finding safer alternatives. EOs, with their ability to inhibit key carbohydrate-digesting enzymes such as α-amylase and α-glucosidase, offer a promising natural solution with bioactive potential (Sharma et al., 2024).

This study is premised on the hypothesis that a complementary blend of EOs can enhance enzyme inhibition more effectively than individual oils, providing a multi-targeted and efficient strategy for managing glucose metabolism. The plants selected for this investigation were false yellowhead, *Inula viscosa* L. (synonym of *Dittrichia viscosa* L. Greuter), rose geranium, *Pelargonium graveolens* L'Hér., and lemongrass, *Cymbopogon citratus* (DC) Stapf, chosen for their well-documented ethnopharmacological significance in treating metabolic disorders and their ability to inhibit key enzymes.

These plants are known for their distinct and complementary chemical profiles, which are believed to interact effectively when combined. The aerial parts of these plants were specifically used due to their high concentration of volatile bioactive metabolites, such as terpenoids and phenolic metabolites, which are commonly associated with enzyme inhibition. These plant parts are also readily available and compatible with conventional extraction techniques, making them sustainable and practical resources for further study.

This paper explores the antidiabetic potential of these EOs. It examines the mechanisms through which their key bioactive metabolite interact with metabolic pathways and cellular receptors involved in blood glucose regulation. The mechanisms of action include inhibition of digestive enzymes responsible for glucose absorption, regulation of insulin signaling pathways, and reduction of inflammation associated with insulin resistance.

In addition to exploring the biological activity of these EOs, the study employs a statistical mixture design to identify optimal EO combinations that maximize enzyme inhibition while minimizing potential side effects. Previous studies on EO combinations have demonstrated that well-formulated blends can enhance the efficacy of complementary metabolites, particularly in antibacterial and antioxidant applications (Fadil et al., 2018; Chraibi et al., 2023). This study extends these findings to the antidiabetic domain, an area that has been comparatively less explored.

To further elucidate the molecular mechanisms underlying enzyme inhibition, this study integrates Density Functional Theory (DFT) calculations and molecular docking simulations to provide deeper insights into the molecular mechanisms underlying enzyme inhibition, helping to assess the efficacy of the identified metabolites for antidiabetic treatment. Researchers increasingly rely on computational approaches, including global reactivity descriptors derived from DFT and molecular docking simulations (Er-Rajy et al., 2023a; Er-rajy et al., 2023d). These tools are used to analyze the electronic properties of the target molecules, enhance understanding of their molecular interactions, assess their potential efficacy, and predict the antidiabetic effects of the identified metabolites.

For the first time, this study evaluates the combined antidiabetic effect of EOs from *I. viscosa*, *P. graveolens*, and *C. citratus* on the inhibitory activities of pancreatic α-amylase and intestinal α-glucosidase, using a centred mixing design. The DFT calculations provided insights into the electrophilic nature of the key metabolites, while molecular docking simulations demonstrated their strong binding affinities to the target enzymes, often surpassing the inhibitory potential of acarbose, a standard antidiabetic drug.

## 2 Materials and methods

### 2.1 Plant material and extraction

**FIGURE 1:**
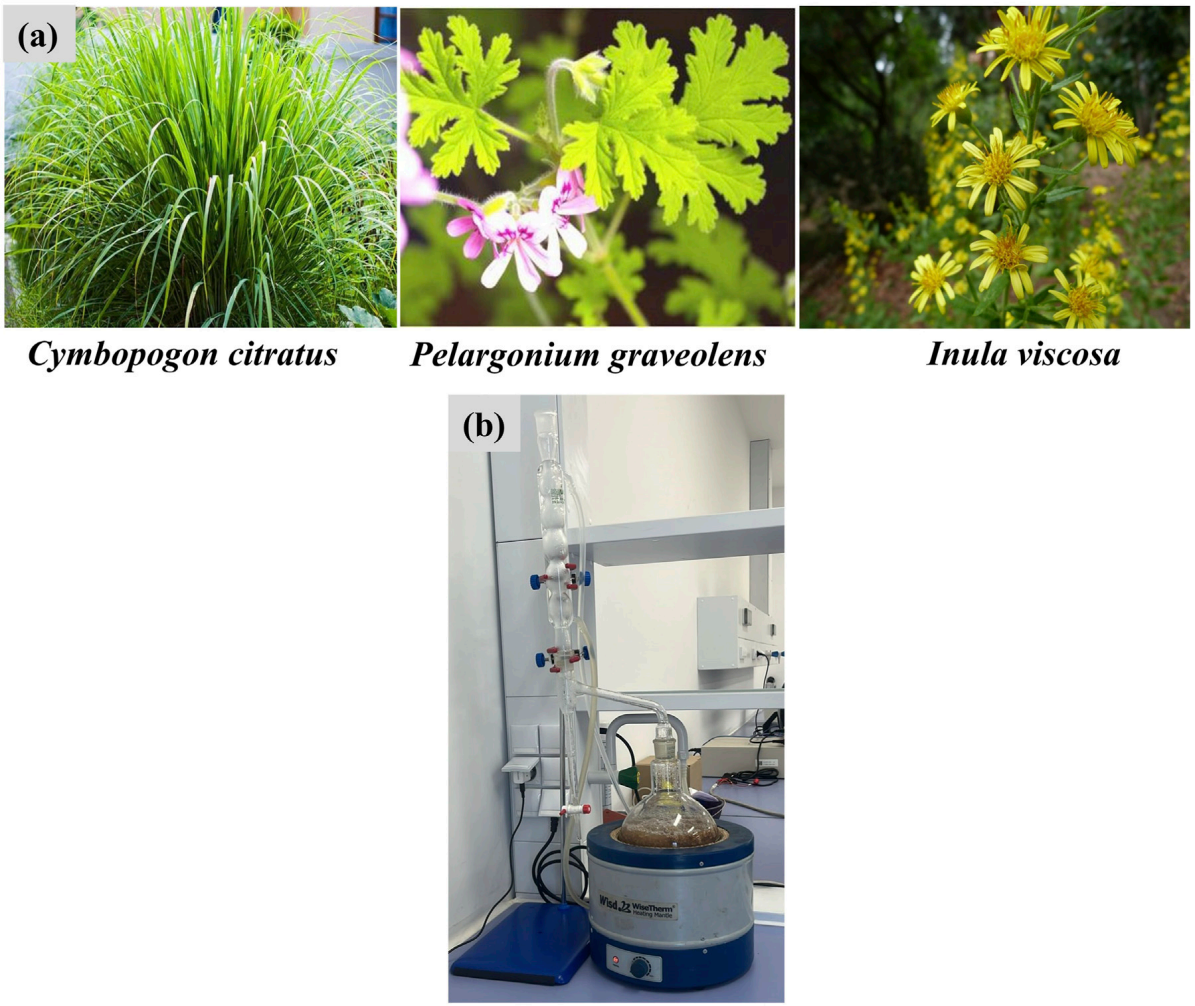
Plant species utilized in this study **(A)** and the hydrodistillation procedure using a clevenger apparatus **(B)**.

Aerial parts of *Inula viscosa*, *Pelargonium graveolens*, and *Cymbopogon citratus* (Figure 1) were harvested in May 2024 from Taourirt, northeastern Morocco (coordinates 34°24′26.32″N, 2°53′50.35″W). The plants were taxonomically identified by Professor Mohamed ADDI, a specialist botanist and researcher at the Laboratory for Improvement of Agricultural Production, Biotechnology, and Environment (LAPABE), Faculty of Sciences, Mohammed Premier University, Oujda, Morocco. Voucher specimens (CLP-007, CLP-008, and CLP-009) were assigned to *I. viscosa*, *P. graveolens*, and *C. citratus*, respectively, and deposited in the herbarium of the Faculty of Sciences, Oujda.

The selection of these plant species was guided by their documented ethnopharmacological relevance in managing metabolic disorders. Traditional knowledge and literature research highlighted their historical medicinal use and distinct chemical profiles, making them suitable candidates for this study. The chemical composition of the EOs was thoroughly analyzed to validate their potential for enzymatic inhibition. This study complies with the Access and Benefit-Sharing (ABS) regulations under the Nagoya Protocol, as applicable in Morocco.

Fresh plant material was collected in sterile plastic bags, transferred to the laboratory, and cut into small fragments. The samples were then dried in a dark, well-ventilated space, and the EOs were extracted by hydrodistillation (Loukili et al., 2023; Taibi et al., 2024d; Elbouzidi et al., 2024b; Laaroussi et al., 2022). Specifically, 300 g of dried plant material was distilled for 180 min using a Clevenger-type apparatus. The extracted oils were collected, dehydrated using anhydrous sodium sulfate, filtered, and stored at 4°C for subsequent analysis.

### 2.2 Headspace procedure

Before isolating the volatile metabolites, 0.5 g of the selected plant material was weighed. The samples were carefully transferred into 20 mL vials using forceps and sealed until they reached room temperature, particularly for frozen samples. Solid-phase microextraction fibers were exposed to the headspace of these samples for 10 min at 80°C. The extracted analytes were desorbed and separated (Loukili et al., 2024b). The extracts were desorbed, separated, and rapidly identified using gas chromatography and mass spectrometry (GC-MS).

### 2.3 Phytochemical characterization of EOs

The phytochemical profile of the EOs was characterized using a Shimadzu GC system (Kyoto, Japan) equipped with a BPX25 capillary column (30 m, 0.25 mm I.D., and 0.25 µm film thickness), consisting of 5% diphenyl and 95% dimethylpolysiloxane, and coupled to a QP2010 mass spectrometer. Helium gas (99.99%) was used at a flow rate of 1.69 L/min. The injection port, ion source, and interface temperatures were set to 250°C. The column temperature was initially held at 50°C for 1 min, then raised to 250°C at a rate of 10°C/min and maintained at 250°C for an additional minute. The sample metabolites were ionized in electron impact mode at 70 eV, and mass spectra were recorded at 40–300 m/z. Each EO sample was injected in a volume of 1 µL in split mode.

The metabolites were identified by comparing their retention times and mass spectra with reference libraries, including the National Institute of Standards (NIST) database (https://webbook.nist.gov/). To further confirm their identification, the retention indices of the detected metabolites were calculated using the conventional Kovats retention index method (Hazarika et al., 2020). Data processing and analysis were performed using LabSolutions software (version 2.5, Shimadzu, Kyoto, Japan) (Haddou M. et al., 2024; Azghar et al., 2023; Haddou et al., 2023c; Kadda et al., 2022).

### 2.4 Inhibition of pancreatic α-amylase *in vitro*.

The *in vitro* inhibition of pancreatic α-amylase was assessed using a modified method as described in the literature (Ouahabi et al., 2024; Taibi et al., 2024b; Bouslamti et al., 2023). The assay mixtures contained 200 µL of phosphate buffer solution (0.02 M, pH = 6.9) and 200 µL of α-amylase enzyme solution. EO solutions at varying concentrations (0.062, 0.125, 0.25, 0.5, and 1 mg/mL) were added and incubated for 10 min at 37°C. Following this, 200 µL of a 1% starch solution was added to each tube, and the reaction mixtures were incubated for 15 min at 37°C. The enzymatic reaction was halted by adding 600 µL of 3,5-dinitro salicylic acid reagent, and the tubes were heated at 100°C for 8 min. After cooling in an ice water bath, 1 mL of distilled water was added to dilute the mixtures. Absorbance was measured at 540 nm using a spectrophotometer. A blank reaction was generated by replacing the EO with phosphate buffer to represent 100% enzyme activity. Acarbose, a known inhibitor, was used as a positive control. The inhibition rate was calculated using the following Equation 1:

$$\text{Inhibition }\left(\%\right)=\left(\frac{\text{AC}-\text{AS}}{\text{AC}}\right)\times 100$$ (1)

where AC represents the absorption of the control reaction (without inhibitor), and AS is the absorbance in the presence of extracts or acarbose.

The IC_50_ value, representing the concentration of the EO that causes 50% inhibition of α-amylase enzyme activity, was determined graphically by plotting the inhibition percentage against the logarithm of EO concentration (Haddou et al., 2023b).

### 2.5 Evaluation of intestinal α-glucosidase inhibition *in vitro*.

The inhibitory activity of intestinal α-glucosidase was assessed following a modified method described by (Elrherabi et al., 2023). The reaction mixtures consisted of 1 mL of phosphate buffer solution (pH = 7.5), 0.1 mL of α-glucosidase enzyme solution (10 IU), and 200 µL of EO solutions at varying concentrations (0.062, 0.125, 0.25, 0.5, and 1 mg/mL). Control samples included distilled water as the negative control and acarbose as the positive control. After pre-incubation at 37°C for 20 min, 0.1 mL of sucrose solution was added to initiate the reaction. After a 5-min incubation at 100°C, the reaction was stopped, and 1 mL of glucose oxidase-peroxidase reagent was added. The mixtures were incubated at 37°C for 10 min, and absorbance was measured at 500 nm. The inhibition rate was calculated using the following Equation 2:

$$\text{Inhibition}\left(\%\right)=\left(\frac{\text{AC}-\text{AS}}{\text{AC}}\right)\times 100$$ (2)

where AC represents the absorption of the control reaction (without inhibitor), and AS is the absorbance in the presence of extracts or acarbose.

The concentration of EO that inhibits 50% (IC_50_) of α-amylase enzyme activity is determined graphically by plotting inhibition percentage against the logarithm of EO concentration.

### 2.6 Mixture design

An augmented simplex-centroid design was conducted to optimize the formulation based on three EOs. This type of experimental design is particularly suited for studying mixtures where the sum of the metabolite proportions remains constant. The augmented simplex-centroid design offers a comprehensive exploration of the mixture space, incorporating points from the classic simplex centroid along with additional axial points (Fadil et al., 2018). This configuration allows for more accurate modelling of non-linear effects and complex interactions between the metabolites. The design enhances the ability to detect curvatures in the response surface by including axial points, thus providing a better understanding of the relationships between mixture composition and antidiabetic activity.

### 2.7 Mixture composition table

As shown in Table 1, each of the three EOs can vary between 0 and 1, ensuring that the sum of their proportions always equals 1. This represents a mixture without constraints (Fadil et al., 2022).

**TABLE 1:** Identification of the formulation of the essential oils.

Essential oil	Coded variable	Level (−)	Level (+)
*Pelargonium graveolens*	X1	0	1
*Inula viscosa*	X2	0	1
*Cymbopogon citratus*	X3	0	1
Sum of proportions		1	

### 2.8 Experimental matrix

**FIGURE 2:**
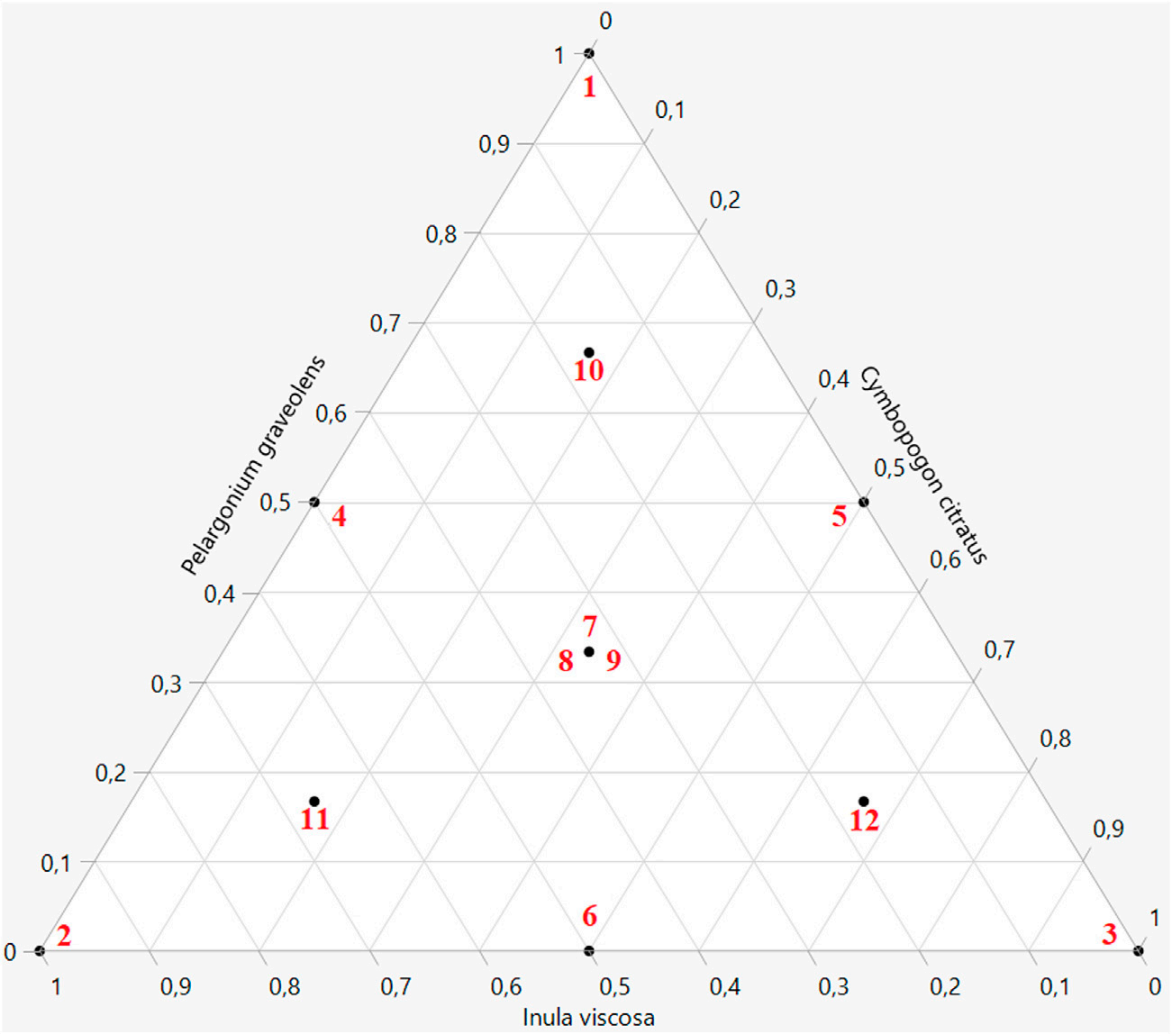
Illustration representing the augmented simplex-centroid design.

The augmented simplex-centroid design was developed to optimize the exploration of the experimental space while maintaining high statistical efficiency, as illustrated in Figure 2. This design comprises 12 trials, structured at 10 unique experimental points, complemented by two replications at the centroid of the mixture triangle. This configuration ensures comprehensive coverage of the formulation space, including the vertices (representing pure EOs), the midpoints of the edges (binary mixtures in equal proportions), the centroid (balanced ternary mixture), and axial points. The replications at the center of the experimental domain allow for the estimation of pure experimental error variance, thus enhancing the robustness of the statistical analysis and improving the accuracy of predictive models (Fadil et al., 2022).

### 2.9 Selected mathematical model

A special cubic model was selected to model the relationship between the proportions of EOs and their antidiabetic activity (Chraibi et al., 2023). This third-order polynomial model is particularly well-suited for mixture designs, as it captures linear effects, binary interactions, and potentially significant ternary interactions. The following equation expresses the general form of the model Equation 3:

$$Y=\alpha_{1}X_{1}+\alpha_{2}X_{2}+\alpha_{3}X_{3}+\alpha_{12}X_{1}X_{2}+\alpha_{13}X_{1}X_{3}+\alpha_{23}X_{2}X_{3}+\alpha_{123}X_{1}X_{2}X_{3}+ɛ$$ (3)

*Y* represents the measured response, in this case, the antidiabetic activity, quantified by IC_50_ values for α-glucosidase and α-amylase, expressed in mg/mL. α₁, α₂, α₃ are the coefficients of the linear terms, representing the primary effects of each EO. α₁₂, α₁₃, α₂₃ are the coefficients of the binary interaction terms, capturing specific interaction between pairs of EOs, while α₁₂₃ is the coefficient of the ternary interaction term, reflecting the combined effect of all three EOs. ε represents the error term, encompassing the variability not explained by the model.

### 2.10 Statistical analysis

The significance of the fitted models was assessed using an F-test for the analysis of variance (ANOVA), which involved calculating the F-ratio (MS_R_/MS_r_) the ratio between the mean square due to regression (MS_R_) and the mean square due to residuals (MS_r_). Model adequacy was evaluated using the ratio of mean square lack of fit (MS_LOF_) to mean square pure error (MS_PE_), denoted as F_ratioLOF/PE_, where higher values indicate a lack of fit. The coefficient of determination (R^2^) assessed the model’s quality, while the significance of the estimated coefficients was determined using Student’s t-test. All statistical tests were conducted at a 95% confidence level. Experimental design conception, statistical analysis, and graphical representation were performed using JMP software V.16 and Design-Expert V.12. Results are presented as mean ± standard deviation (SD).

### 2.11 Optimization tools

Optimal oil formulations were determined using contour and surface plots based on iso-response curves. The desirability function was then employed to identify the precise optimal settings with a compromise percentage. This function assigns values between 0 and 1, where 0 represents factor levels leading to an unacceptable response, and 1 indicates the maximum desired response. This approach enabled the quantification of the optimal adjustment accuracy within the defined range (Fadil et al., 2024).

### 2.12 DFT approach

The local and global reactivity of organic and inorganic metabolites in various fields, including synthesis, medicine, and corrosion, has been studied using several theoretical approaches (Chérif et al., 2024). DFT has been particularly crucial in examining these aspects. In this study, DMol³ techniques were employed with the double numerical plus polarization basis set and generalized gradient approximation with the Perdew–Burke–Ernzerhof functional (GGA/PBE) within Materials Studio software to optimize the structure of the EO metabolites and two key proteins (PDB: 1B2Y and PDB: 2QMJ). Using Equations 4–10, we computed the following theoretical parameters: highest occupied molecular orbital (HOMO) and lowest unoccupied molecular orbital (LUMO) energies, energy gap (ΔE_gap_), electron affinity (EA), ionization potential (IP), electronegativity (χ), electron transfer percentage (ΔN), and dipole moment (μ) characteristics (Attarki et al., 2023; Mansour et al., 2024a).

$${∆E}_{gap}=E_{LUMO}‐E_{HOMO}=\text{IP}‐\text{EA}$$ (4)

$$\text{IP}=-E_{HOMO}$$ (5)

$$\text{EA}=-E_{LUMO}$$ (6)

$$\chi_{inh}=\frac{IP+EA}{2}$$ (7)

$$\eta_{inh}=\frac{{∆E}_{gap}}{2}$$ (8)

$$\mu =\frac{E_{LUMO}+E_{HOMO}}{2}$$ (9)

$$\Delta N_{\max}=\frac{-\mu }{\eta_{inh}}$$ (10)

Furthermore, these theoretical instruments facilitated the identification of several isosurfaces, including the HOMO and LUMO, Non-Covalent Interactions (NCI), and Reduced Density Gradient (RDG), using Multiwfn, VMD, and Gnuplot software (Ling et al., 2023). Additionally, the interactions between the main volatile metabolites of EOs (MVCEO) and the two proteins (PDB: 1B2Y and PDB: 2QMJ) were investigated by optimizing their structures using the Forcite module in Materials Studio software (Mansour et al., 2024b).

### 2.13 Molecular docking

Before initiating the docking study, the most abundant metabolites in the mixture were visualized using ChemDraw 16.0 (Mills, 2006), and their geometry was optimized using the MM2 method. The Molecular Operating Environment (MOE) software (Vilar et al., 2008) was employed to analyze ligand-protein receptor interactions. This included removing water molecules, correcting missing side-chain residues, adding non-polar hydrogens, performing molecular docking, and visualizing the outcomes. The targeted proteins were sourced from the RCSB Protein Data Bank (RCSB PDB) (https://www.rcsb.org/) (Noguchi and Akiyama, 2003), which houses thousands of genetic targets.

In preparation for molecular docking, hydrogen atoms were added to the most prominent metabolites in the mixture, and surrounding water molecules were removed (Er-Rajy et al., 2023b; Er-rajy et al., 2023c). The potential energy was then calculated by incorporating atomic charges and other parameters, adjusted using the MMFF94x force field. Finally, the metabolites were saved in MDB format for docking (Er-rajy et al., 2023c).

To perform molecular docking, we used two receptors extracted from the Protein Data Bank (PDB ID: 2QMJ and 1B2Y). For 2QMJ, the grid was defined with coordinates X = −20.807 Å, Y = −6.586 Å, and Z = −5.073 Å, while for 1B2Y, the grid parameters were X = 18.909 Å, Y = 5.790 Å, and Z = 47.006 Å. In both cases, the grid dimensions were set to 60 × 60 × 60 points along the X, Y, and Z directions (Nahoum et al., 2000; Sim et al., 2008). The molecular docking simulations were performed using a semi-flexible model, as we defined the target pocket for both proteins while keeping the ligands flexible.

In this study, three metabolites (2-camphol acetate, 3,7-dimethyl-2-octen-1-ol, and citronellol) were selected due to their pronounced antidiabetic potential. The primary objective was to evaluate their inhibitory activity against two key enzymes, α-glucosidase and α-amylase, which play crucial roles in carbohydrate metabolism and are therapeutic targets for managing diabetes (Loukili et al., 2022a; Thilagam et al., 2013).

## 3 Results and discussion

### 3.1 Phytochemical characterization of EOs

**FIGURE 3:**
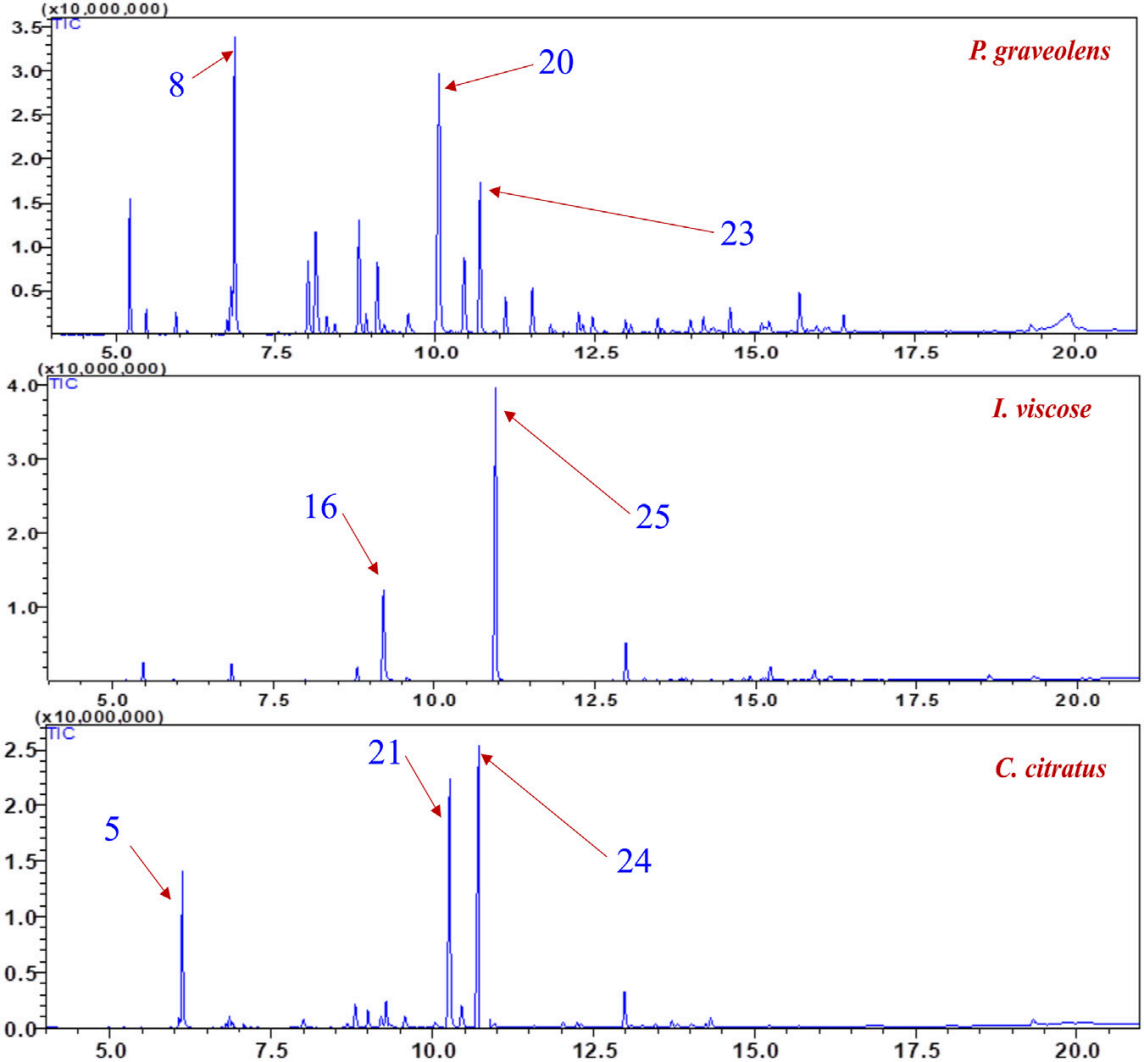
GC-MS profile of essential oils of *Inula viscosa*, *Pelargonium* graveolens, and *Cymbopogon citratus*.

Each plant’s EO chromatograms were compared to reference metabolites from a spectral library and information database. The GC-MS system provided mass chromatograms and retention indices for the various substances potentially present in the extract. Figure 3 and Table 2 illustrate the chromatographic profiles of each plant’s EO. The GC-MS analysis of the EO from *P. graveolens* identified 32 metabolites.

**TABLE 2:** Composition of essential oils from *Inula viscosa*, *Pelargonium graveolens*, and *Cymbopogon citratus* profiled by GC-MS.

N°	Metabolite	RI^a^	RI^b^	Area %		
				*P. graveolens*	*I. viscosa*	*C. citratus*
1	α-Pinene	957	927	5.37	0.16	0.11
2	Camphene	966	945	1.1	2.81	-
3	β-Pinene	981	980	1	0.28	-
4	5-Hepten-2-one, 6-methyl-	984	985	-	-	0.77
5	β-Myrcene	986	991	-	-	15.82
6	p-Cymene	1025	1026	0.56	-	-
7	1,4-Dimethyl-4-vinylcyclohexene	1026	949	2.5	-	-
8	Eucalyptol	1027	1028	13.4	2.48	0.94
9	β-Linalool	1082	1098	4.01	-	1.17
10	α-Thujone	1089	1102	6.93	-	-
11	β-Thujone	1095	1114	0.96	-	-
12	trans-Rose oxide	1101	1126	0.63	-	-
13	Camphor	1116	1143	6.27	2.49	3.06
14	p-Menthan-3-one	1121	1128	1.1	-	-
15	Verbenol	1124	1121	3.85	-	2.35
16	Camphol	1132	1165	0.78	19.32	1.56
17	Carane, 4,5-epoxy-, trans	1135	1179	-	-	2.7
18	p-Menth-1-en-4-ol	1137	1177	-	-	0.88
19	α-Terpieol	1145	1192	1.46	1.08	2.9
20	2-Octen-1-ol, 3,7-dimethyl-	1162	1463	-	-	0.98
21	Citronellol	1247	1228	18.67	-	-
22	2-Isopropenyl-5-methylhex-4-enal	1254	-	-	-	29.25
23	Lemonol	1260	1255	5.08	-	2.62
24	α-Citral	1269	1247	8.12	-	24.7
25	2-Camphanol acetate	1277	1285	-	51.12	-
26	Linalool, formate	1281	1215	1.99	-	-
27	(−)-Myrtenyl acetate	1294	1326	2.46	-	-
28	Geranic acid	1310	1355	1.16	-	1.32
29	α-Copaene	1317	1377	0.56	-	0.63
30	α-Bourbonene	1322	1385	1.22	-	-
31	β-Caryophyllen	1336	1418	0.83	7.46	3.9
32	β-Farnesene	1349	1458	0.92	-	0.61
33	α-Muurolene	1354	1499	-	0.71	1.05
34	Prenyl benzoate	1359	1453	-	0.66	-
35	β-Selinene	1360	1463	-	0.66	-
36	Geranyl propionate	1362	1430	1.01	-	0.81
37	Varidiflorene	1367	-	1.04	-	0.55
38	2-Octen-1-ol, 3,7-dimethyl-	1377	1463	1.43	-	1.32
39	Linalyl iso-valerate	1384	1484	0.69	0.99	-
40	Isoaromadendrene epoxide	1570	1594	0.85	3.15	-
41	β-Cedren-9-,α,-ol	1583	1645	2.78	2.94	-
42	Globulol	1587	1583	-	1.31	-
43	Nerolidyl acetate	1588	1687	-	0.82	-
44	γ-Eudesmol	1592	1600	1.27	-	-
45	Aromadendrene oxide-(1)	1629	1613	-	1.56	-

^a^ : Kovats retention index calculated from alkanes series on the MS, capillary column (C6–C24).

^b^ : Kovats index-Retention index from data libraries (NIST; https://webbook.nist.gov/cgi/cbook.cgi?ID=C29050337&Units=SI&Mask=2000#Gas-Chrom).

The GC-MS analysis identified 32 metabolites in *P. graveolens*, with eucalyptol (13.30%), citronellol (18.67%), 2-octen-1-ol, 3,7-dimethyl (8.12%), α-thujone (6.93%), and camphor (6.27%), which together make up more than half of the total composition. In *I. viscosa*, 18 metabolites were found, with 2-camphanol acetate being the most abundant (51.12%), followed by Camphol (19.32%) and β-caryophyllen (7.46%). *C. citratus*, contained 23 metabolites, with α-citral (24.70%) and two-isopropenyl-5-methylhex-4-enal (29.25%) and β-myrcene (15.82%) as key components.

A study by Ainane et al. investigated the chemical composition of EOs extracted from *P*. *graveolens* collected in the Khenifra region, Morocco. The EO, obtained through hydrodistillation of the dried aerial parts and analyzed by GC-MS, revealed geraniol (31.01%), citronellol (29.52%), linalool (10.50%), citronellyl formate (9.06%), geranyl formate (5.75%), and iso-menthone (2.86%) as the primary metabolites (Ainane et al., 2021). Similarly, Aissa et al. reported that the major metabolites of *I. viscosa* EO were (Z)-neryl isovalerate (17.5%–29.8%), 1,10-di-epi-cubenol (19.1%–27.2%), and 2,5-dimethoxy-p-cymene (5.9%–17.7%) (Aissa et al., 2019). For *C. citratus*, GC-MS analysis identified key metabolites, including 7,7-dimethyl-4-methylenebicyclo [4.1.0]heptan-3-ol (15.07%) and trans-3,7-dimethyl-2,6-octadienal, commonly known as α-citral (42.27%) (Naqqash and Al-Bazaz, 2019).

The volatile metabolites found in various aromatic plants and extracted as EOs are frequently employed for their medicinal properties, including their potential antidiabetic effects (Hammouti et al., 2023). These oils may enhance enzyme activity associated with glucose metabolism, combat oxidative stress, improve blood glucose management, and decrease blood sugar by increasing insulin sensitivity and reducing insulin resistance (Taibi et al., 2023).

Terpenes (monoterpenes and sesquiterpenes), phenolic metabolites, alcohols, aldehydes, ketones, esters, and oxides comprise the most complex combinations of volatile organic molecules that make up EOs. Each element adds to the oil’s distinct scent and therapeutic qualities. For instance, the phenolic eugenol possesses analgesic and antibacterial qualities, while the monoterpene limonene is well-known for its anti-inflammatory and antioxidant qualities. Due to their ability to successfully repel mosquitoes and other pests, lemongrass and geraniol are incredibly well-liked natural insect repellents (Hazarika et al., 2020). Other substances known to have soothing and anxiolytic qualities, such as linalool and α-terpineol, are frequently used in aromatherapy to encourage relaxation.

EOs’ bioactivity is ascribed to both the interactions between their metabolite chemicals and the specific characteristics of each one. The intricate chemistry of EOs gives rise to this interaction, as different metabolite combinations either intensify therapeutic effects or reduce possible toxicity (Vaou et al., 2022). For instance, phenolic substances like thymol and carvacrol are frequently more effective when paired with terpene hydrocarbons, although the interaction of monoterpenes with alcohols might increase antibacterial activity. EOs are more effective and have a more comprehensive range of medicinal uses because of their complex composition, making them useful in pharmaceutical and industrial settings (Haddou m. et al., 2024).

### 3.2 Headspace analysis

**FIGURE 4:**
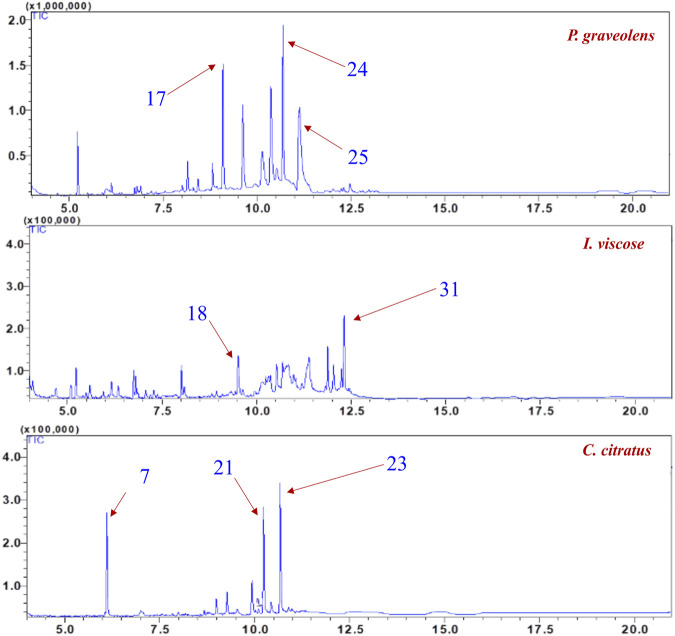
Chemical metabolites and their relative percentages in the essential oils of *Inula viscosa*, *Pelargonium graveolens*, and *Cymbopogon citratus* based on headspace analysis.

Table 3 and Figure 4 displayed the concentration of volatile metabolites in *I. viscosa*, *P. graveolens*, and *C. citratus* as determined through headspace analysis. Table 3 revealed the presence of various metabolites such as heptanal, α-pinene, β-myrcene, p-cymene, D-limonene, and others. The concentration of these metabolites differs across the three plants, reflecting their unique volatile compositions.

**TABLE 3:** Volatile metabolites of *Inula viscose*, *Pelargonium graveolens* and *Cymbopogon citratus* using headspace.

N°	Metabolite	RI^a^	RI^b^	Area %		
				*G. rosat*	*I. viscosa*	*C. citratus*
1	Heptanal	890	910	-	0.68	-
2	Benzaldehyde	920	926	-	1.25	-
3	α-Pinene	929	927	3.09	4	-
4	Camphene	948	945	-	0.29	-
5	2-Heptenal, (Z)-	980	964	-	2.64	-
6	β-Pinene	1012	980	-	0.16	-
7	β-Myrcene	986	991	0.88	3.22	17.95
8	Octanal	1000	983	-	2.64	-
9	p-Cymene	1022	1026	0.1	2.88	-
10	D-Limonene	1025	1031	0.18	2.57	-
11	Eucalyptol	1030	1028	0.59	-	2.1
12	Linalyl alcohol	1083	1098	1.2	4.14	
13	α-Thujone	1089	1126	2.45	-	-
14	Rose oxide	1096	1124	0.08	-	-
15	β-Ocimene	1101	1233	1.35	-	-
16	6-Octenal, 3,7-dimethyl-	1117	1152	2.72	0.47	-
17	Verbenol	1123	1153	11.45	-	4.77
18	Azulene	1143	1311	8.79	6.83	-
19	Pulegone	1158	1244	2.61	-	9.23
20	2-Octen-1-ol, 3,7-dimethyl-	1165	1463	9.11	-	23.87
21	2-Propenal, 3-phenyl-	1258	1266	12.2	-	-
22	2-Decenal, (E)-	1263	1255	4.64	6.02	-
23	β-Citral	1268	1240	-	7.59	28.42
24	Citronellyl formate	1268	1275	15.67	4.02	-
25	2-Undecanone	1278	1291			-
26	Carvacrol	1283	1298	19.31	2.79	-
27	2,5,5-Trimethylcyclohex-2-enone	1285	-	-	2.61	-
28	2,4-Decadienal	1290	1284	-	7.7	-
29	α-Cubebene	1305	1351	-	5.98	-
30	(2E)-2-Undecenal	1310	1371	-	5.27	4.17
31	α-Ylangene	1316	1350	-	4.58	-
32	Copaene	1318	1377	0.9	10.48	-
33	Caryophyllene	1336	1485	-	0.71	2.05
34	Isothujol	1347	1170	-	6.9	1.2
35	Germacrene D	1358	1480	0.25	3.58	
36	Crocetane	1417	1813	1.37	-	-
37	Heptadecane, 8-methyl-	1427	1738	1.37	-	-

^a^ : Kovats retention index calculated from alkanes series on the MS, capillary column (C6–C24).

^b^ : Kovats index-retention index from data libraries (NIST; https://webbook.nist.gov/cgi/cbook.cgi?ID=C29050337&Units=SI&Mask=2000#Gas-Chrom) and (Fadil et al., 2018).

The analysis revealed that in *P. graveolens*, 2-propenal, 3-phenyl- (12.2%), carvacrol (19.31%), and 6-octen-1-ol, 3,7-dimethyl-, formate (15.67%) were present in relatively high amounts, suggesting these as potential main metabolites. In *I. viscosa*, significant metabolites included 2.6-octadienal, 3.7-dimethyl-, (E)- (7.59%), 2-decenal, (E)- (6.02%), and copaene (10.48%). The volatile profile of *C. citratus* was dominated by β-citral (28.42%) and 3,7-dimethyl-2-Octen-1-ol (23.87%).

Some metabolites were found exclusively in specific plants. For example, the azulene is present in *P. graveolens* and *I. viscosa* but not in *C. citratus*. Similarly, the thujone concentration in *P. graveolens* was 0.08%. In contrast, no thujone was detected in *I. viscosa* or *C. citratus*, making *P. graveolens* the plant with the lowest reported concentration of this metabolite.

Understanding the unique chemical compositions of these plants aids in characterizing their distinct properties. For example, certain metabolites contribute to each plant’s specific fragrance, which may be important for applications related to scent, flavor, or medicinal characteristics. By comparing the profiles of the three plants, we can identify both similarities and differences in their volatile components, providing valuable insights for further exploration in aromatic and therapeutic applications.

#### 3.3 α-amylase inhibition *in vitro*

Pancreatic α-amylase (EC 3.2.1.1) is a key enzyme that catalyzes the hydrolysis of α-1,4 glycosidic bonds in starch, amylopectin, amylose, glycogen, and various maltodextrins. It is essential in breaking down starch to facilitate digestion (Sharma et al., 2008). While large molecules like starch cannot pass the blood-brain barrier, α-amylase breaks it down into smaller sugars that can pass. However, if excessive starch is converted into sugar, it can lead to elevated blood sugar levels. This, in turn, triggers insulin release to help cells metabolize and store the excess glucose as glycogen. This balance is maintained in healthy individuals, but excessive α-amylase activity, combined with insulin deficiency or resistance, can lead to hyperglycemia. Therefore, inhibiting α-amylase has been a research focus to control high blood sugar levels (Sharma et al., 2008).

**FIGURE 5:**
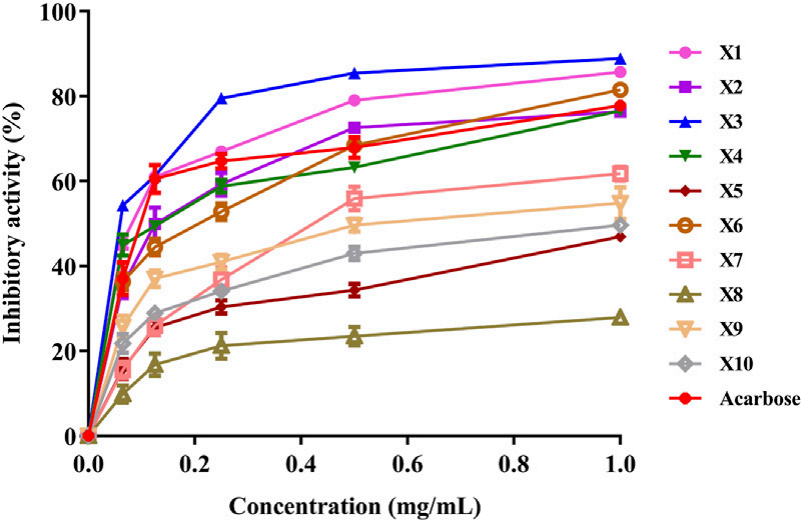
Evaluation of α-amylase inhibition by various essential oil fractions of *Inula viscosa*, *Pelargonium graveolens*, and *Cymbopogon citratus*.

Testing the *in vitro* effects of extracts from three different plants on α-amylase activity (Figure 5) and their mixtures at varying percentages revealed that *C. citratus* (X3) is the most potent inhibitor, with an IC₅₀ value of 0.044 ± 0.002 mg/mL, requiring the lowest concentration to achieve 50% enzyme inhibition. *P. graveolens* (X1) follows with an IC₅₀ of 0.053 ± 0.003 mg/mL, showing strong inhibitory activity. *I. viscosa* (X2) has the highest IC₅₀ (0.067 ± 0.015 mg/mL), making it the least effective of the three, though it still outperforms the standard control, acarbose, which has an IC₅₀ of 0.089 ± 0.008 mg/mL. These results suggest that all three plants, especially *C. citratus*, are more effective α-amylase inhibitors than acarbose, highlighting their potential as natural alternatives for managing α-amylase activity and related conditions such as diabetes.

Few studies have examined the inhibitory effect of *C. citratus* on α-amylase. One study showed that this plant’s α-amylase inhibitory activity (AAI) remained relatively stable across different pre-treatment methods. Drying methods slightly reduced AAI, while spray drying with maltodextrin resulted in a 40% decrease in AAI (Widiputri et al., 2017). Another study revealed that organic extracts of *I. viscosa* effectively inhibit α-amylase, with the methanolic extract of *I. viscosa* exhibiting the highest inhibitory (IC₅₀ = 22.3 μg/mL), surpassing acarbose (IC₅₀ = 33.0 μg/mL) (Asraoui et al., 2021). Similarly, the EO of *P. graveolens* demonstrated dose-dependent inhibition of α-amylase, with an IC₅₀ value of 66.09 ± 1.43 μg/mL, compared to acarbose’s lower IC₅₀ value of 6.64 ± 0.32 μg/mL (Afifi et al., 2013; Jaradat et al., 2022).

When evaluating α-amylase inhibition from various plant extract combinations, the mixture of *P. graveolens* and *I. viscosa* (X4) exhibited the most potent inhibitory effect, with an IC₅₀ value of 0.036 ± 0.004 mg/mL, surpassing acarbose (IC₅₀ = 0.089 ± 0.008 mg/mL). Other notable combinations include X9, which predominantly contains *I. viscosa* (IC₅₀ = 0.054 ± 0.004 mg/mL), and X10, where *C. citratus* is the major metabolite (IC₅₀ = 0.050 ± 0.018 mg/mL). These findings suggest that specific plant extract mixtures, especially those incorporating *P. graveolens*, offer superior α-amylase inhibition compared to acarbose. The complementary action observed in these combinations indicates their potential as more effective natural alternatives for managing postprandial hyperglycemia.

In traditional medicine, the idea of complementary action is important because it highlights the improved pharmacological effects that occur when several metabolites cooperate rather than act alone (Hoeng et al., 2011). Network pharmacology reveals that interactions between different metabolites and their targets can result in more effective treatments with fewer side effects than single-target drugs (Jaradat et al., 2022). This holistic approach, rooted in traditional medicine, acknowledges that various active metabolites in a formula or plant can act on multiple targets simultaneously, enhancing bioavailability and therapeutic outcomes. The success of traditional medicine in clinical practice suggests that developing new drugs should focus on the complementary effects of combined metabolites, optimizing efficacy while minimizing adverse effects. This strategy aligns with traditional medicine principles and leverages modern scientific advancements to improve drug discovery and development (Yuan et al., 2017).

#### 3.4 α-glucosidase inhibition *in vitro*

**FIGURE 6:**
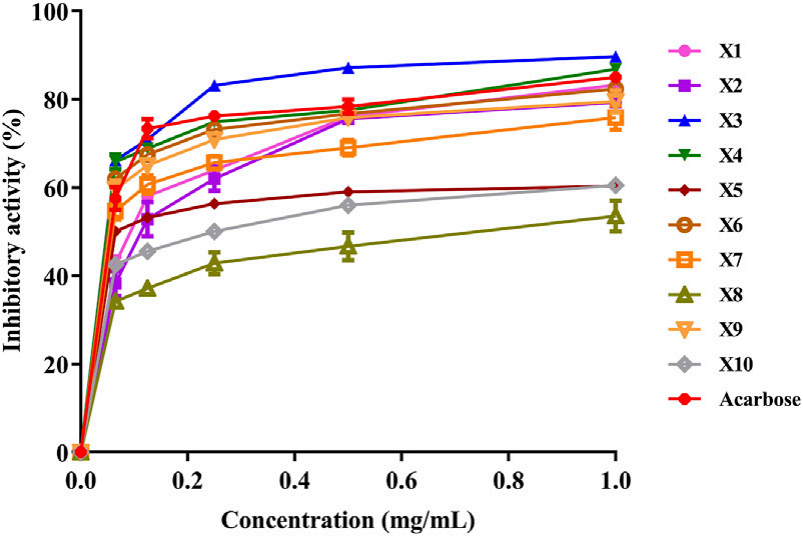
Evaluation of α-glucosidase inhibition by various essential oil fractions of *Inula viscosa*, *Pelargonium graveolens*, and *Cymbopogon citratus*.

α-glucosidase is a crucial enzyme involved in carbohydrate digestion, making it an important target in developing hypoglycemic drugs. It catalyzes the hydrolysis of α-1,4-glucosidic bonds, breaking oligosaccharides like maltose and sucrose in the small intestine (Zhang et al., 2022; Tian et al., 2021). The results of α-glucosidase inhibition (Figure 6) showed that *C. citratus* (X3) exhibits the most potent inhibitory activity, with an IC_50_ value of 0.044 ± 0.002 mg/mL, lower than that of acarbose (IC_50_ = 0.059 ± 0.009 mg/mL). This indicates that *C. citratus* is more effective than acarbose in inhibiting α-glucosidase activity. Previous studies have demonstrated that this plant exhibit significant inhibition of α-glucosidase activity, attributed to its high flavonoid content (Santoso et al., 2018; Gunawan-Puteri et al., 2020).

*P. graveolens* (X1) also showed notable inhibitory effects, with an IC_50_ of 0.068 ± 0.002 mg/mL, while *I. viscosa* (X2) demonstrated the weakest inhibition of the three plants, with an IC_50_ of 0.078 ± 0.018 mg/mL. Despite the fact that *I. viscosa* exhibits moderate activity, it is less effective than acarbose. Previous studies on *P. graveolens* leaves also reported notable inhibition on α-glucosidase, with an IC_50_ of 93.72 ± 4.76 μg/mL comparable to acarbose (IC_50_ = 80.4 ± 2.17 μg/mL). This suggests that *P. graveolens* can potentially manage carbohydrate digestion and metabolic disorders such as diabetes (Aydin et al., 2022). Similarly, prior studies on *I. viscosa* leaves confirmed their significant inhibitory effects against α-glucosidase, underscoring their potential for modulating carbohydrate digestion (Aydin et al., 2022). The results suggest that *C. citratus* has the highest potential for α-glucosidase inhibition, outperforming acarbose.

The inhibition results for various plant mixtures revealed notable variations. Among the tested mixtures, X4 (a 50/50 mix of *P. graveolens* and *I. viscosa*) and X5 (a 50/50 mix of *P. graveolens* and *C. citratus*) exhibited comparable inhibition, with IC_50_ values of 0.046 ± 0.002 mg/mL and 0.043 ± 0.002 mg/mL, respectively. These mixtures demonstrated significant α-glucosidase inhibitory activity, with X5 showing the lowest IC_50_, indicating the highest potency among the tested combinations.

Mixture X6, a 50/50 mix of *I. viscosa* and *C. citratus*, had an IC_50_ of 0.048 ± 0.002 mg/mL, slightly higher than X4 and X5 but still effective compared to the control. Mixtures X8 (66.67% *P. graveolens*, 16.67% *I. viscosa*, and 16.67% *C. citratus*) and X10 (16.67% *P. graveolens*, 16.67% *I. viscosa*, and 66.67% *C. citratus*) demonstrated IC_50_ values of 0.053 ± 0.002 mg/mL and 0.048 ± 0.002 mg/mL, respectively, indicating moderate inhibitory effects.

The X7 mixture, which combined equal proportions of all three plants (33.33% each), showed consistent inhibition, with IC_50_ values of 0.046 ± 0.004, 0.046 ± 0.015, and 0.045 ± 0.008 mg/mL across three repetitions. This suggests a balanced combination of the three extracts maintains effective α-glucosidase inhibition. Compared to acarbose (IC_50_ = 0.059 ± 0.009 mg/mL), most plant mixtures, especially X5 and X4, exhibited comparable or superior inhibitory activity. This indicates that these plant combinations may serve as effective alternatives to acarbose for managing α-glucosidase activity.

The results highlight the importance of complementary plant metabolites in α-glucosidase inhibition. The varying degrees of inhibition across the different plant mixtures suggest that interactions between *P. graveolens*, *I. viscosa*, and *C. citratus* contribute to their overall effectiveness. For instance, mixtures of *P. graveolens* with either *I. viscosa* or *C. citratus* (X4 and X5) exhibited more powerful inhibitory effects than the individual extracts, with lower IC_50_ values. This indicates that combining these plants enhances their collective ability to inhibit α-glucosidase.

It is well known that combining metabolites with varying properties can lead to interactions that produce effects distinct from those observed with individual metabolites. These interactions may be synergistic, complementary, potentiating, antagonistic, or additive. From a pharmacological perspective, the combined effects observed in mixtures of extracts with different compositions are particularly noteworthy (Ouahabi et al., 2024). Such combinations can result in biological effects greater than the sum of their individual metabolites, enhancing the therapeutic potential of the combined extracts (Ouahabi et al., 2023).

Our *in vitro* experiments demonstrate the complementary action, as illustrated in Figures 5, 6. These figures highlight the optimization of EO combinations for inhibiting α-amylase and α-glucosidase activities using a statistical mixture design approach. The figures clearly show how specific EO ratios, identified as optimal blends, significantly enhance enzyme inhibition compared to individual oils. These results reinforce EO combinations as a natural diabetes treatment option by highlighting their pharmacological relevance in targeting important enzymes involved in glucose metabolism.

### 3.5 Experimental design

A simplex-centroid design was employed to investigate various combinations of the EOs under study. Table 4 presents the experimental results for each EO combination, including the observed IC_50_ responses for both α-glucosidase and α-amylase inhibition. To minimize bias, the experiments were conducted in a randomized order. Each reported response value represents the mean of three replicate measurements.

**TABLE 4:** IC_50_ values for α-glucosidase and α-amylase for various essential oil combinations.

*Fraction*	*P. graveolens*	*I. viscosa*	*C. citratus*	IC_50_ α-glucosidase	IC_50_ α-amylase
X1	1	0	0	0.068 ± 0.003	0.047 ± 0.008
X2	0	1	0	0.078 ± 0.008	0.067 ± 0.004
X3	0	0	1	0.045 ± 0.002	0.034 ± 0.004
X4	0.5	0.5	0	0.044 ± 0.001	0.022 ± 0.003
X5	0.5	0	0.5	0.043 ± 0.005	0.055 ± 0.006
X6	0	0.5	0.5	0.047 ± 0.001	0.047 ± 0.002
X7	0.333	0.333	0.333	0.045 ± 0.002	0.152 ± 0.007
X8	0.333	0.333	0.333	0.047 ± 0.003	0.15 ± 0.003
X9	0.333	0.333	0.333	0.045 ± 0.009	0.161 ± 0.008
X10	0.667	0.167	0.167	0.053 ± 0.006	0.09 ± 0.006
X11	0.167	0.667	0.167	0.052 ± 0.001	0.1 ± 0.004
X12	0.167	0.167	0.667	0.043 ± 0.002	0.09 ± 0.003

### 3.6 Statistical analysis and model validation

ANOVA was performed to assess the proposed models’ relevance and goodness of fit for the two responses: IC_50_ of α-glucosidase and IC_50_ of α-amylase (Table 5).

**TABLE 5:** Statistical relevance and model fit for α-glucosidase and α-amylase IC_50_ responses.

Model	DF	IC50 α-glucosidase				IC50 α-amylase			
		SSE	MS	F	p-value	SSE	MS	F	p-value
Regression	6	0.001322	0.00022	60.8477	0.0002	0.02526	0.00421	114.64	<0.0001
Residual	5	0.000018	0.000004			0.00018	0.000037		
Lack of fit	3	0.000015	0.000005	3.8582	0.2126	0.00011	0.000038	1.12	0.5047
Pure error	2	0.000003	0.000001			0.00007	0.000034		
Total	11	0.001340				0.02544			
R^2^		0.98				0.99			

For α-glucosidase IC_50_, the main regression effect was highly significant (p = 0.0002), indicating a strong relationship between the independent variables (EO proportions) and the response. Similarly, for α-amylase IC_50_, the regression effect showed even more pronounced significance (p < 0.0001), reinforcing the overall validity of the models for both responses. Both models exhibited no lack of fit, with p-values well above the 0.05 threshold (p = 0.21 for α-glucosidase and p = 0.5 for α-amylase), indicating that the models accurately capture the variability of the data without overfitting.

**FIGURE 7:**
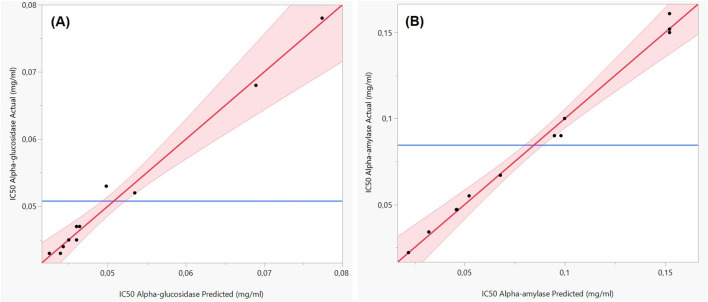
Plots of observed values *versus* predicted values for the IC₅₀ responses of α-glucosidase **(A)** and α-amylase **(B)**.

The obtained R^2^ was remarkably high: 0.98 for α-glucosidase IC_50_ and 0.99 for α-amylase IC_50_. These values demonstrate excellent agreement between experimental and predicted values, suggesting that the models explain 98% and 99% of the variance in the responses, respectively. The graphical representation of observed *versus* predicted values (Figure 7) further supports these results. The alignment of the data points along the regression line for both responses visually confirms the models’ strong predictive accuracy and quality of fit.

Together, these statistical analyses validate the relevance and reliability of the mathematical models developed to predict the inhibitory activity of EO mixtures on α-glucosidase and α-amylase. This provides a solid basis for optimizing the antidiabetic formulations based on EO combinations.

### 3.7 Factor effects and mathematical models

A comprehensive analysis of the effects of the studied factors was conducted, incorporating t-student statistical values and corresponding *p*-values. These results are synthesized in Table 6, which provides an overview of the significance of each model term.

**TABLE 6:** Estimated regression coefficients for the models.

Term	Coefficient	IC_50_ α-glucosidase		IC_50_ α-amylase	
		Estimation	p-value	Estimation	p-value
*Pelargonium graveolens*	α1	0.069	<0.0001*	0.046	0.0005*
*Inula viscosa*	α2	0.077	<0.0001*	0.068	<0.0001*
*Cymbopogon citratus*	α3	0.045	<0.0001*	0.032	0.0028*
P. graveolens *I. viscosa	α12	−0.115	<0.0001*	−0.140	0.0051*
P. graveolens *C. citratus	α13	−0.052	0.0025*	0.052	0.1362
I. viscosa*C. citratus	α23	−0.059	0.0014*	−0.016	0.6011
P. graveolens *I. viscosa*C. citratus	α123	0.200	0.0106*	3.110	<0.0001*

*Statistically significant

For the α-glucosidase IC_50_ response, statistical analysis revealed the significance of the following components: (i) linear terms (α₁, α₂, and α₃) represent the main effects of each EO; (ii) binary interaction terms (α₁₂, α₁₃, and α₂₃) indicate specific interaction between pairs of oils; and (iii) the ternary interaction term (α₁₂₃) highlights the combined effect of all three EOs. The following equation expresses the mathematical model for α-glucosidase IC_50_:

$$Y_{IC50-\alpha \;glucosidase}=0.069\;X_{1}+0.077X_{2}+0.045X_{3}-0.11X_{1}X_{2}-0.05X_{1}X_{3}-0.059X_{2}X_{3}+0.2X_{1}X_{2}X_{3}+ɛ$$

Regarding the α-amylase IC_50_ response, the analysis highlighted the significance of the following terms: (i) linear terms (α₁, α₂, and α₃) confirming the individual influence of each EO; (ii) binary interaction term (α₁₂), suggesting a specific interaction between two oils; (iii) the ternary interaction term (α₁₂₃) indicating the complex effects of combining all three oils. The established mathematical model for α-amylase IC_50_ is represented by the following equation:

$$Y_{IC50\;\alpha \;amylase}=0.046\;X_{1}+0.068X_{2}+0.032X_{3}-0.14X_{1}X_{2}+3.11X_{1}X_{2}X_{3}+ɛ$$

These mathematical models, derived from rigorous statistical analysis, provide a solid foundation for predicting and optimizing the inhibitory activity of EO mixtures on α-glucosidase and α-amylase. The presence of significant interaction terms in both models underscores the complexity of the relationships between components and justifies the use of a mixture design in this study.

### 3.8 Responses optimization and desirability analysis

The study aimed to optimize the formulation of EOs to maximize the inhibitory activity of α-glucosidase and α-amylase. The optimization was performed using iso-response profiles and the desirability function.

#### 3.8.1 Optimization of α-glucosidase inhibition

**FIGURE 8:**
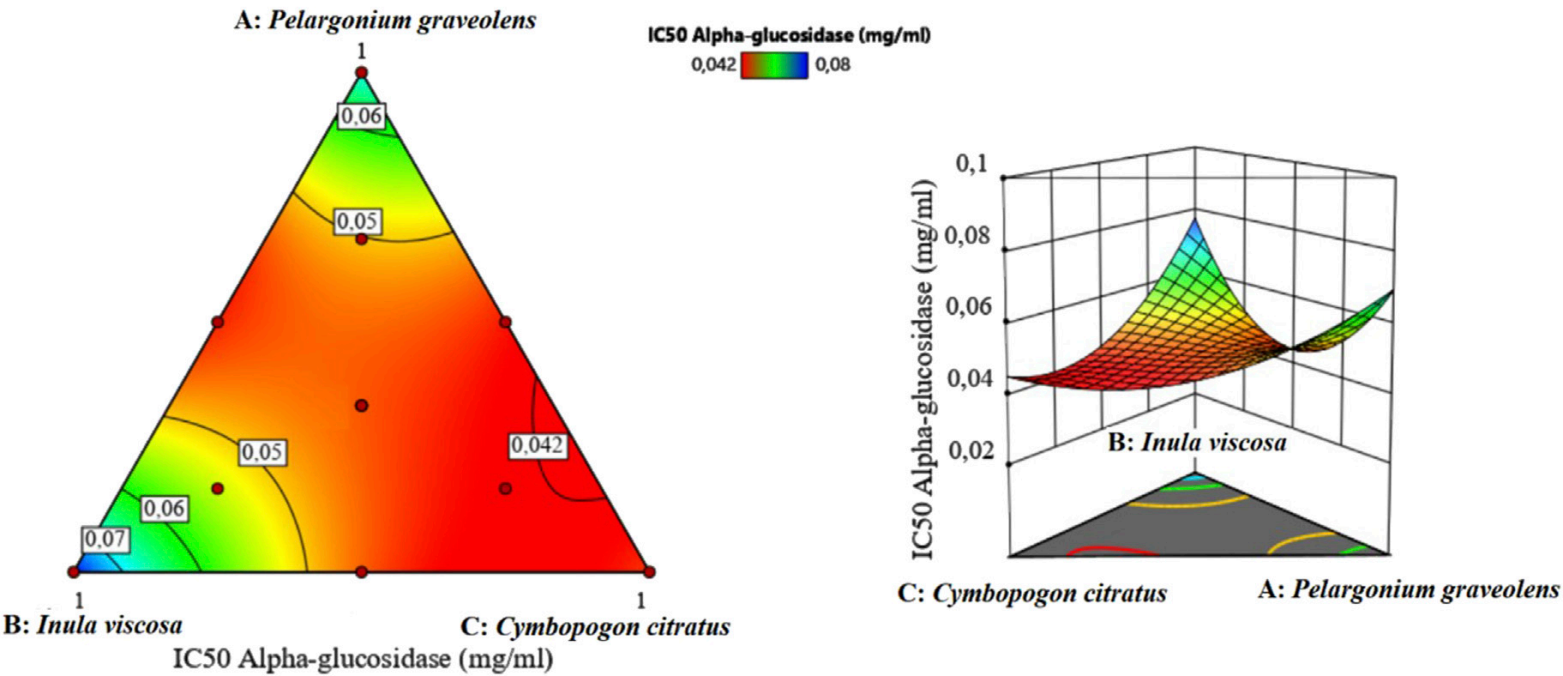
2D and 3D mixture plots showing the optimal zone for α-glucosidase IC_50_ response.

A comprehensive analysis of 2D and 3D mixture graphs, as illustrated in Figure 8, provided valuable insights into the optimal formulation for α-glucosidase inhibition. The analysis revealed that a very low IC_50_ value of approximately 0.042 mg/mL could be achieved, indicating potent enzyme inhibition. This optimization pointed to a binary mixture predominantly composed of two EOs: *P. graveolens* and *C. citratus*.

**FIGURE 9:**
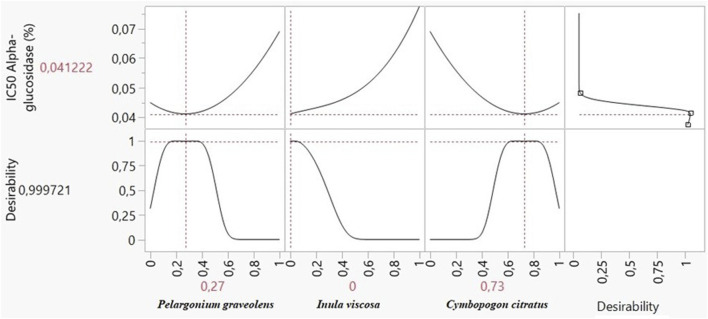
Desirability profile of optimal conditions for minimizing the α-glucosidase IC_50_ response.

Further refinement using the desirability function, a powerful statistical tool for multi-response optimization, allowed for precise determination of the optimal mixture composition to minimize the IC_50_ value. The results indicated an optimal binary mixture consisting of 73% *C. citratus* and 27% *P. graveolens*, predicted to achieve an even lower IC_50_ value of 0.041 mg/mL for α-glucosidase inhibition (Figure 9).

Notably, the desirability score for this optimized formulation reached 99%, reflecting a very high confidence in the predicted outcome and underscoring the robustness of the optimization process. This near-perfect desirability score indicates that the proposed mixture closely approaches the theoretical ideal for α-glucosidase inhibition.

#### 3.8.2 Optimization of α-amylase inhibition

**FIGURE 10:**
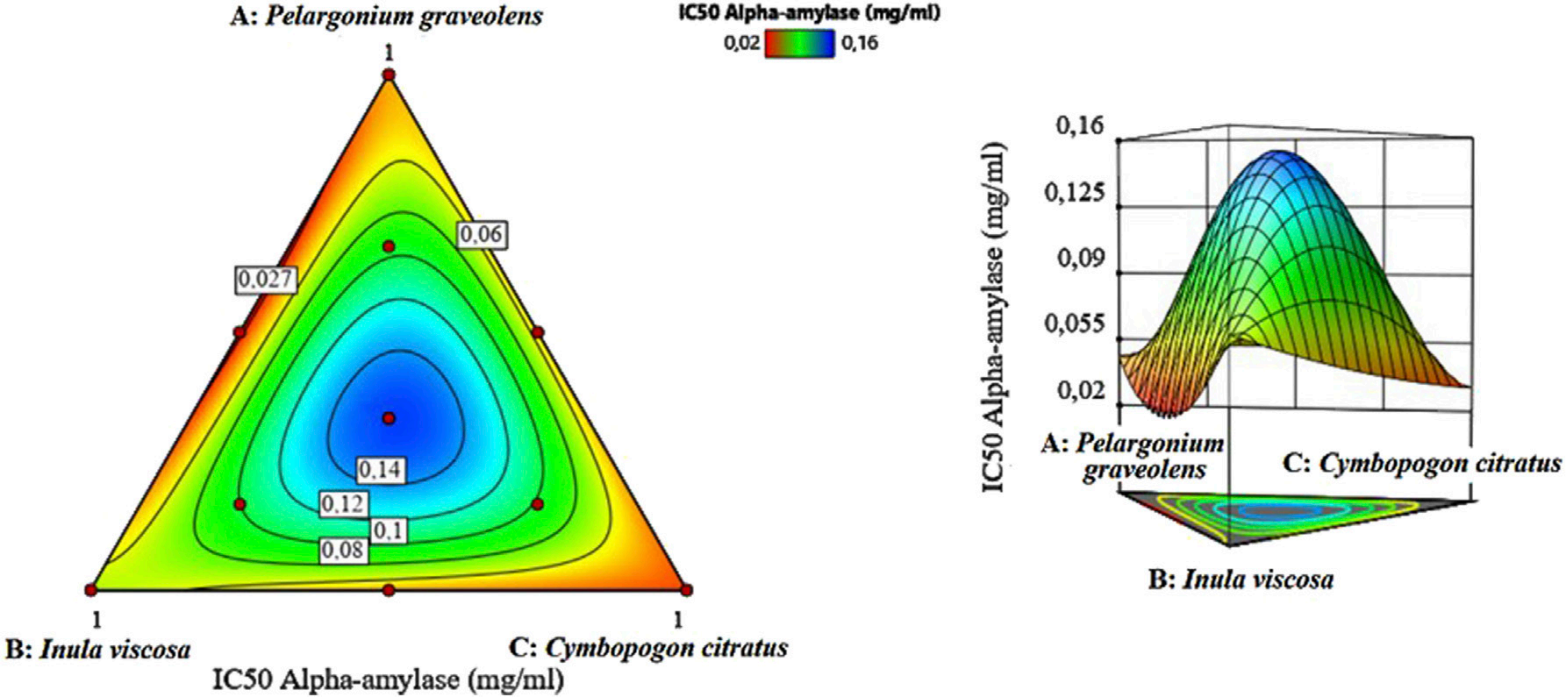
2D and 3D mixture plots showing the optimal zone for the α-amylase IC_50_ response.

The potential for inhibiting α-amylase was assessed using 2D and 3D mixture graphs (Figure 10). The visual analysis suggested an optimal IC_50_ value of approximately 0.022 mg/mL, indicating potent enzyme inhibition. The optimal binary mixture consisted of *P. graveolens* and *I. viscosa* EOs.

**FIGURE 11:**
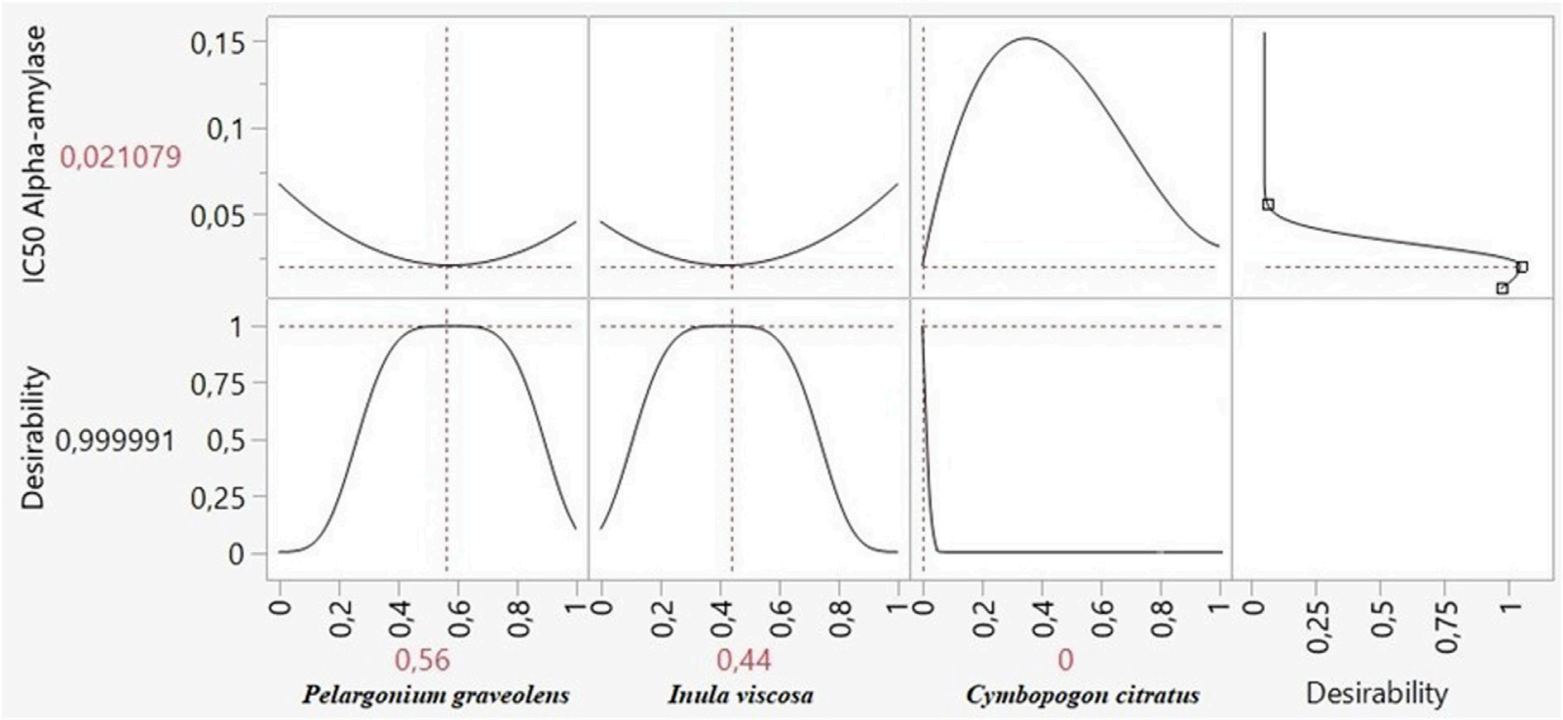
Desirability profile of optimal conditions for minimizing the α-amylase IC_50_ response.

The desirability function analysis further refined the optimal mixture composition to 56% *P. graveolens* and 44% *I. viscosa*, predicted to achieve an IC_50_ of 0.021 mg/mL for α-amylase inhibition, improving upon the initial graphical estimate (Figure 11). The desirability score for this optimized formulation also reached 99%, indicating exceptional confidence in the predicted outcome.

These findings highlight the complementary action of combining *P. graveolens* and *I. viscosa* EOs in a specific ratio to maximize α-amylase inhibition. Identifying this optimal mixture could lead to the development of effective natural α-amylase inhibitors, which could potentially be applied in managing conditions like type 2 diabetes and obesity, where carbohydrate digestion plays a crucial role.

### 3.9 Global and local reactivity results

**FIGURE 12:**
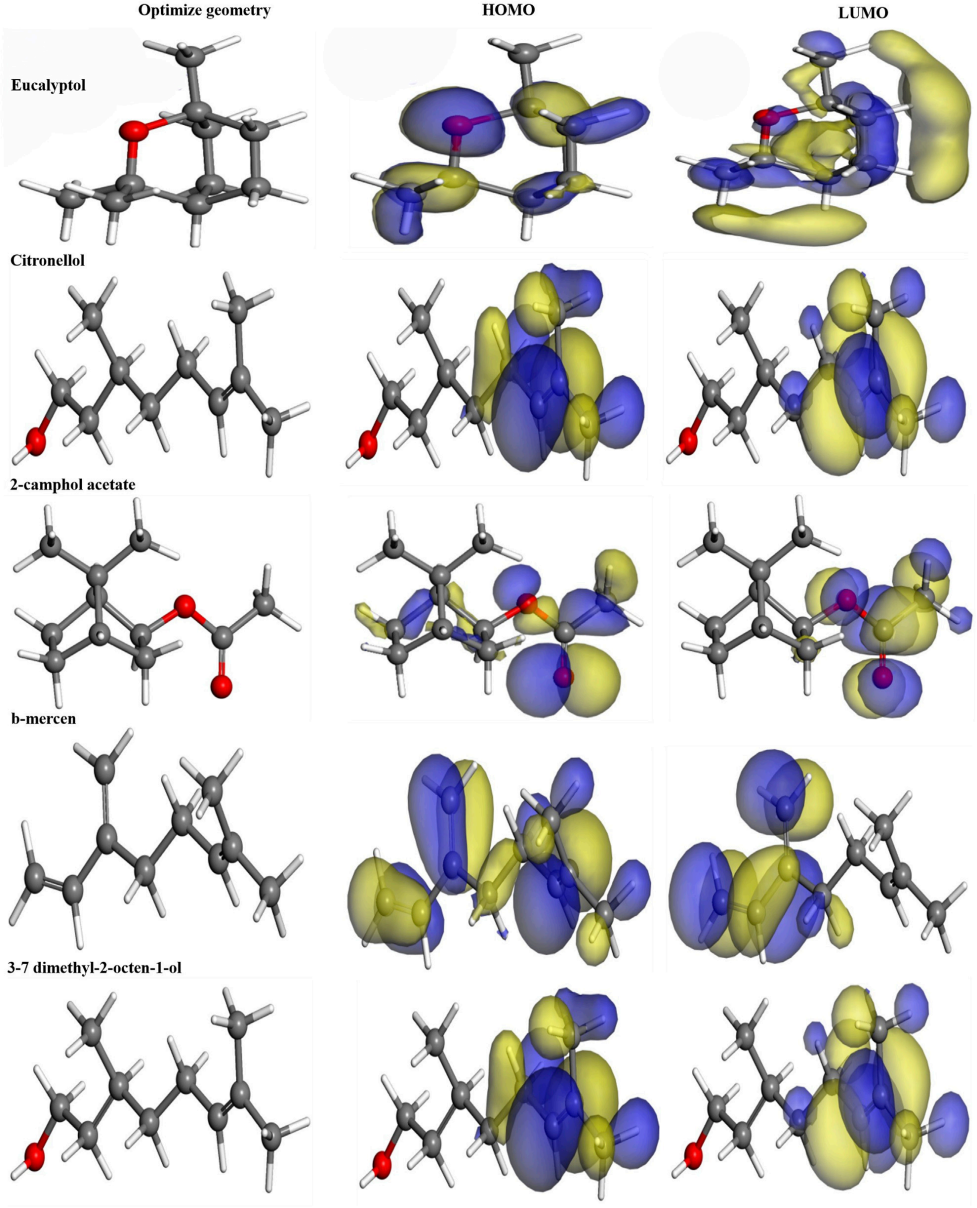
Optimized structure, HOMO, and LUMO of the main volatile metabolites of essential oils derived using the DFT/GGA tool.

DFT is an essential tool for deriving significant isosurfaces like the HOMO and LUMO and for calculating both local and global reactivity using numerous quantum parameters. These findings are depicted in Figure 12, which shows the optimized geometry, HOMO, and LUMO of the main volatile metabolites of EOs (MVCEO). As indicated by the HOMO and LUMO isosurfaces, the electron distribution in MVCEO is centred around the benzene ring, which serves as the electron cloud’s active centre (Hsu et al., 2024). This is confirmed by the positive electron transfer number (ΔNmax) in Table 7, indicating that the molecule is electrophilic and prefers to receive rather than lose electrons from other species.

**TABLE 7:** Chemical quantum parameters of the main volatile metabolites of essential oils were established using the DFT/GGA theoretical model. All parameters are in electronvolts (eV).

Parameter	E_HOMO_	E_LUMO_	IP	EA	ΔE_gap_	χ_inh_	η_inh_	ΔN_inh_
Eucalyptol	−5.242	1.161	5.242	−1.161	6.403	2.040	3.201	0.637
Citronellol	−5.121	0.007	5.121	−0.007	5.128	2.557	2.564	0.997
3–7 dimethyl-2-octen-1-ol	−5.122	0.009	5.122	−0.009	5.131	2.556	2.565	0.996
2-camphol acetate	−6.046	−0.653	6.046	0.653	5.393	3.349	2.696	1.242
b-mercene	−5.238	−1.506	5.238	1.506	3.732	3.372	1.866	1.807

### 3.10 Adsorption of MVCEO on 1B2Y and 2QMJ proteins

**FIGURE 13:**
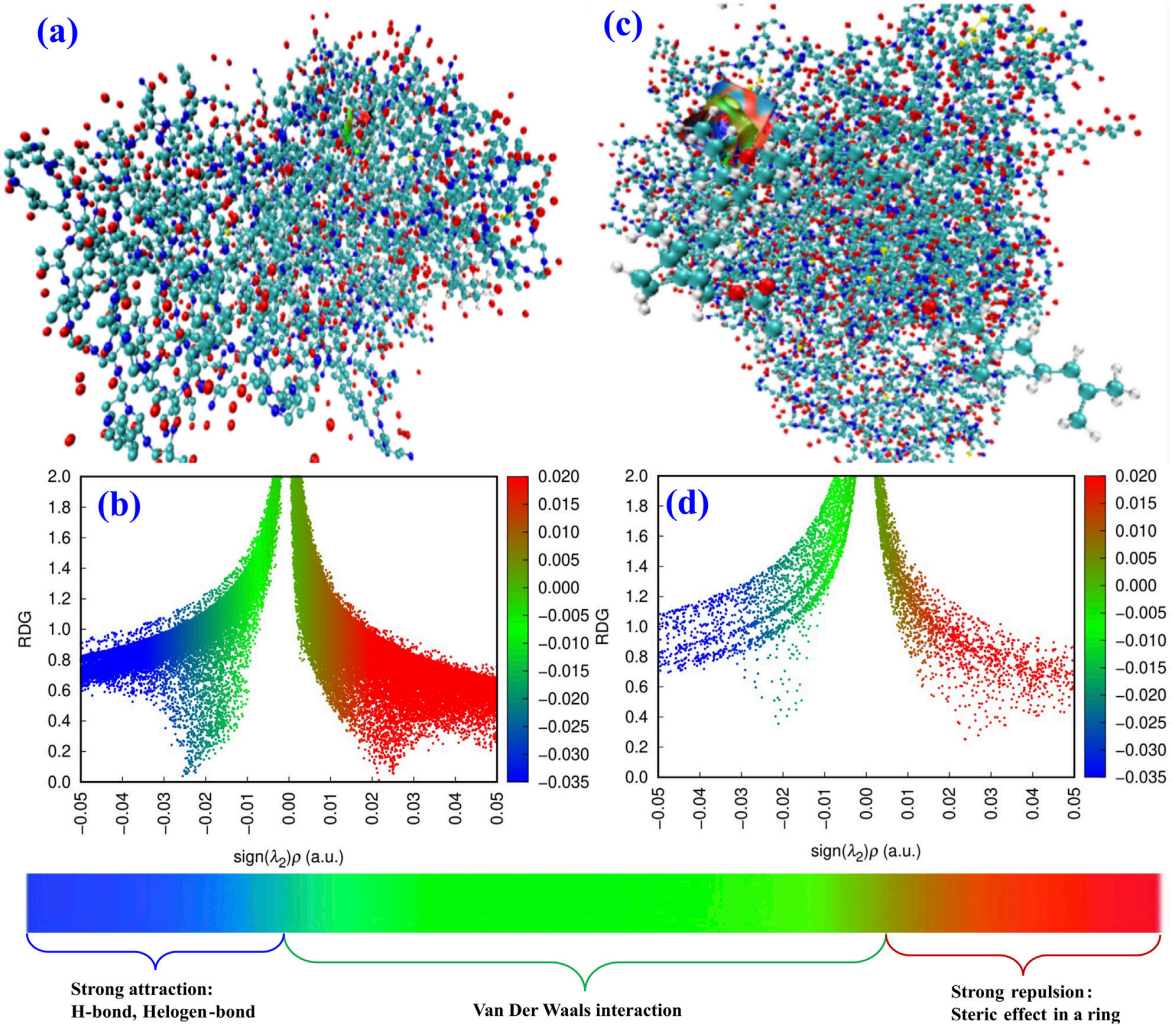
Top view of the NCI **(A)** and RDG **(B)** isosurfaces for α-amylase protein adsorption, and NCI **(C)** and RDG **(D)** for α-glucosidase protein adsorption.

Different local reactivities of MVCEO were found, requiring further investigation into the molecule’s adsorption and interactions with two proteins (PDB: 1B2Y and PDB: 2QMJ). We optimized the structures of MVCEO and 1B2Y and 2QMJ proteins using two theoretical techniques: RDG and NCI, as shown in Figure 13. The Forcite tools in Materials Studio were utilized for optimization, while Gnuplot and Multiwfn software were used to generate the RDG and NCI isosurfaces. The RDG and NCI isosurfaces show blue, green, and red regions, corresponding to Van der Waals interactions and strong attractions (H-bonds) (Al-Otaibi et al., 2023; Sampathkumar et al., 2024). These outcomes validate the experimental findings and demonstrate that MVCEO exhibits local and global reactivity.

### 3.11 Molecular docking

Table 8 summarizes the binding energy scores and specific interactions between the functional groups of the selected metabolites and the target protein residues. The study aimed to simulate the internal dynamics of infected cells after treatment with the proposed inhibitors. Molecular docking was performed using the MOE module between the metabolites 2-camphol acetate, 3,7-dimethyl-2-octen-1-ol, and citronellol, and the PDB co-crystals 1B2Y and 2QMJ, corresponding to α-amylase and α-glucosidase inhibitors, respectively.

**TABLE 8:** Molecular docking results for three receptors.

Metabolite	PDB ID	Receptor	Energy (kcal/mol)	Bonds formed between functional groups of metabolite and protein residues	
				Functional groups	Residues
2-camphol acetate	2QMJ	α-glucosidase	−4.7111	Oxygen	Arg 526, Met 444
3,7 dimethyl-2-octen-1-ol			−4.9326	HO, Carbon	Asp 443, Tyr 299
Citronellol			−4.7179	HO	Arg 526, Met 444, Asp 203
2-camphol acetate	1B2Y	α-amylase	−4.8186	Oxygen	His 299
3,7 dimethyl-2-octen-1-ol			−4.6851	HO	Tyr 67, Ala 128
Citronellol			−4.8839	OH	Arg 195, Asp 300

**FIGURE 14:**
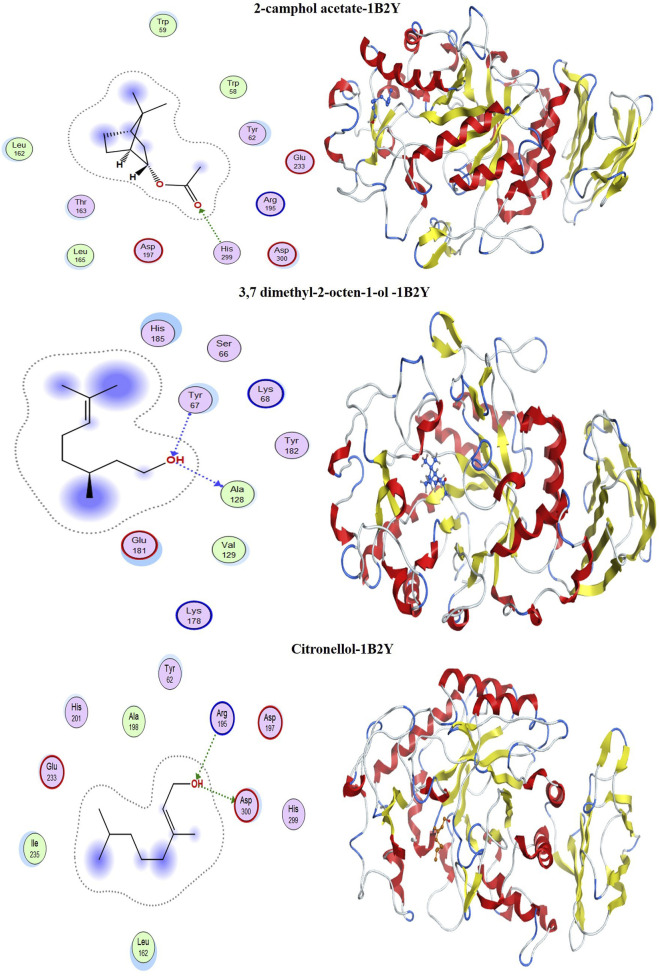
Most significant docking interactions of the metabolites with the α-amylase enzyme.

**FIGURE 15:**
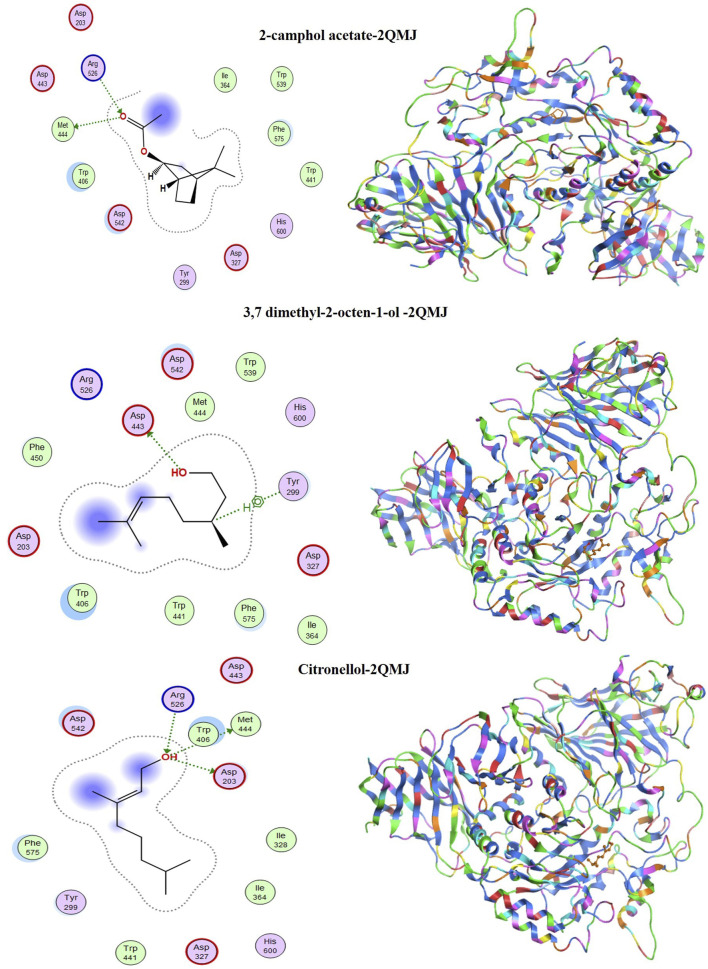
Most significant docking interactions of the metabolites with the α-glucosidase enzyme.

Figures 14, 15 display the docking complex. Table 8 details the ligand types (hydrogen bonding interaction centres), their corresponding protein receptors, and the calculated binding energies for the molecular docking simulation. In the study of three antidiabetic metabolites, the docked molecules exhibited energy score (S) values ranging from −4.8839 to −4.6851 kcal/mol (Table 8), indicating a strong affinity for the target protein 1B2Y. For 2-camphol acetate, a single bond was formed with the His 299 residue. 3,7-dimethyl-2-octen-1-ol interacted with Tyr 67 and Ala 128 residues, while citronellol established two bonds with Arg 195 and Asp 300. These varied interactions highlight the inhibitory potential of these molecules against the target protein 1B2Y, suggesting their role in modulating the enzyme’s activity.

In evaluating the metabolites as potential α-glucosidase inhibitors, the docking energy values ranged from −4.9326 to −4.7111 kcal/mol, indicating stable interactions with the target enzyme 2QMJ. Figure 15 shows that 2-camphol acetate formed two bonds with the Arg 526 and Met 444 residues. 3,7-dimethyl-2-octen-1-ol interacted with Asp 443 and Tyr 299, while citronellol formed three bonds with Arg 526, Met 444, and Asp 203. These diverse interactions highlight the inhibitory potential of these molecules against the target protein 2QMJ. The strong interactions observed between the most abundant molecules in the mixture and the selected receptors suggest that these molecules interact with the targeted proteins. This affinity is crucial for developing effective antidiabetic drugs.

The molecular docking studies provide theoretical insights into the potential binding affinities of the selected metabolites with the carbohydrate digestive enzymes. It is important to note that these findings are purely computational and do not represent a definitive mechanism of action. Instead, they propose potential interactions that could contribute to the enzyme inhibition observed. To fully understand these interactions, further experimental studies are necessary to evaluate binding affinity specifically and uncover the underlying pharmacological mechanisms.

While the findings highlight the potential of EO blends for inhibiting key carbohydrate-digesting enzymes, this study has limitations. The enzymatic assays were conducted *in vitro*, which may not fully replicate the complexity of *in vivo* metabolic processes. Furthermore, the effects of these EO combinations on long-term glucose regulation, potential toxicity, and bioavailability remain unexplored. Addressing these limitations through animal models and clinical studies will be crucial for translating these findings into therapeutic applications.

## 4 Conclusion

In conclusion, this study examined the EO extraction and identification of three medicinal plants *I. viscosa*, *P. graveolens*, and *C. citratus* along with their potent antidiabetic properties. Our research demonstrated that these plants have significant potential in inhibiting key enzymes related to diabetes management, such as α-glucosidase and α-amylase. The analysis revealed that *C. citratus* EO is rich in α-citral, myrcene, and isopropenyl-5-methylhex-4-enal, while *P. graveolens* EO primarily contains eucalyptol and citronellal. The main metabolite of *I. viscos*a EO was identified as 2-camphanol acetate. The study identified the optimal binary mixture for α-glucosidase inhibition, comprising 73% *C. citratus* and 27% *P. graveolens*. This specific combination yielded the lowest IC_50_ value of 0.041 mg/mL with a desirability score of 99%, demonstrating an exceptionally high potential for α-glucosidase inhibition. For α-amylase inhibition, the optimal binary mixture was 56% *P. graveolens* and 44% *I. viscosa*, resulting in an IC_50_ value of 0.021 mg/mL and a similarly high desirability score of 99%. *In silico* studies provided preliminary theoretical insights, suggesting that the major metabolites in these mixtures may exhibit strong binding affinities with the target proteins (1B2Y and 2QMJ), indicating their potential as antidiabetic agents. The interactions between these EOs and the enzymes α-glucosidase and α-amylase highlight the possibility of developing potent, natural inhibitors for diabetes management.

## Data Availability

The raw data supporting the conclusions of this article will be made available by the authors, without undue reservation.
